# Arterial Blood Pressure Is Closely Related to Ascites Development in Compensated HCV-Related Cirrhosis

**DOI:** 10.1371/journal.pone.0095736

**Published:** 2014-04-22

**Authors:** Eduardo Vilar Gomez, Ana Torres Gonzalez, Luis Calzadilla Bertot, Ali Yasells Garcia, Yoan Sanchez Rodriguez, Yadina Martinez Perez

**Affiliations:** 1 Department of Research and Clinical Experimentation, National Institute of Gastroenterology, Havana, Cuba; 2 Department of Hepatology, National Institute of Gastroenterology, Havana, Cuba; University of Modena & Reggio Emilia, Italy

## Abstract

**Background:**

Arterial blood pressure (BP) is a reliable marker of circulatory dysfunction in cirrhotic patients. There are no prospective studies evaluating the association between different levels of arterial BP and ascites development in compensated cirrhotic patients. Therefore, we evaluated the relationship between arterial BP and ascites development in compensated cirrhotic patients.

**Materials and Methods:**

A total of 402 patients with compensated HCV-related cirrhosis were prospectively followed during 6 years to identify ascites development. At baseline, patients underwent systolic, diastolic and mean arterial pressure (MAP) measurements. Any history of arterial hypertension was also recorded. The occurrence of events such as bleeding, hepatocellular carcinoma, death and liver transplantation prior to ascites development were considered as competing risk events.

**Results:**

Over a median of 156 weeks, ascites occurred in 54 patients (13%). At baseline, MAP was significantly lower in patients with ascites development (75.9 mm/Hg [95%CI, 70.3–84.3]) than those without ascites (93.6 mm/Hg [95% CI: 86.6–102.3]). After adjusting for covariates, the 6-year cumulative incidence of ascites was 40% (95%CI, 34%–48%) for patients with MAP<83.32 mm/Hg. In contrast, cumulative incidences of ascites were almost similar among patients with MAP values between 83.32 mm/Hg and 93.32 mm/Hg (7% [95% CI: 4%–12%]), between 93.32 mm/Hg and 100.31 mm/Hg (5% [95% CI: 4%–11%]) or higher than 100.31 mm/Hg (3% [95% CI: 1%–6%]). The MAP was an independent predictor of ascites development.

**Conclusions:**

The MAP is closely related to the development of ascites in compensated HCV-related cirrhosis. The risk of ascites development increases in 4.4 fold for subjects with MAP values <83.32 mm/Hg.

## Introduction

Chronic hepatitis C virus (HCV) infection is a leading cause of end-stage liver disease worldwide [1]. Once HCV-related cirrhosis has developed, the annual risk of clinical decompensation has been estimated at ∼6% (range, 4–8%) per year. [2]–[7] The most common cause of decompensation is ascites, which has been estimated to range between 3 and 5% per annum. [2], [5], [7]–[10]; once present, it has a negative impact on short-term survival. [7], [8], [11] The pathophysiology of ascites has been extensively reviewed elsewhere. [12] In compensated cirrhosis, circulatory dysfunction is a crucial step for ascites development. The derangement of the systemic and splanchnic circulation with a significant reduction of the effective circulating volume leads to the neurohumoral activation with the subsequent retention of sodium and water. [13], [14] In the context of profound circulatory dysfunction, the compensatory increase of the cardiac output is unable to provide adequate support following the reduction of circulatory volume related to peripheral vasodilation, thus contributing to more advanced clinical stages of the disease. [15]–[17].

In clinical practice, arterial hypotension is a reliable marker of circulatory dysfunction in cirrhotic patients. Among patients with ascites, hepatorenal syndrome may occur in the setting of marked circulatory dysfunction, which is characterized by arterial hypotension and activation of neurohumoral systems. [12], [16], [18] A previous study has reported that arterial blood pressure is an independent predictor of survival in cirrhotic patients with ascites. Individuals with a mean arterial pressure <82 mm/Hg had a 1-year survival of 40%, compared to 70% for those >82 mm/Hg. [19].

Recently it has been suggested that arterial hypertension, which is characterized by increased systemic vascular resistance, can counteract the peripheral vasodilation in cirrhotic patients and protect against vasodilatory complications, such as hepatorenal and hepatopulmonary syndrome. [20] Hypertensive cirrhotic patients are hyperdynamic with central hypovolaemia, but have no signs of overall peripheral vasodilatation. A recent study has reported that a history of high blood pressure serves as a protective factor for liver-related mortality and clinical decompensation. [2] However, this study did not focus on the different types of clinical decompensation. Although arterial hypotension has been directly related to a hyperdynamic circulation and consequently to ascites development, there is no prospective study focusing on the long-term effect of low blood pressure (BP) on ascites development. Additionally, the potential role of arterial hypertension as a protective factor for vasodilatory complications such as ascites has not been prospectively investigated. In this study, we have demonstrated the long-term effect of arterial BP levels on ascites development in a prospective cohort of compensated HCV-related cirrhotic patients. We have also evaluated baseline characteristics and their association with the development of ascites.

## Materials and Methods

### Study Design, Participants and Setting

Our group of research has conducted a Cuban national project to define the natural history of HCV-related cirrhosis to describe the frequency and temporal development of liver-related deaths or transplant, and the development of major clinical outcomes of hepatic decompensation. Additionally, we also evaluated the association between baseline characteristics and the development of major clinical outcomes. The results of this study have been published recently. [2] Although the relationship between baseline characteristics and the development of hepatic decompensation was reported, the association with each event of decompensation was not addressed. Thus, more detailed descriptions of subsequent analyses of our study will be reported separately. All of these analyses were planned before the data were collected.

Within the overall study design [2] we performed a prospective, longitudinal study of HCV-related cirrhotic patients who were consecutively evaluated at a tertiary care academic center between January 2004 and June 2007. We enrolled those who fulfilled the following inclusion criteria irrespective of any previous antiviral treatment status (either naïve or non-responders or sustained viral responders): male and female patients >18 years of age, no history of hepatic decompensation, with or without current or previous history of essential hypertension, absence of active alcoholism, and ability to provide informed consent. The diagnosis of cirrhosis was based on liver biopsy (Ishak fibrosis stage 5–6) or a combination of unequivocal clinical data (splenomegaly, spider angiomata, palmar erythema, gynecomastia, and jaundice), and compatible laboratory (thrombocytopenia, leucopenia, hypoalbuminemia and hyperbilirubinemia), ultrasonography (coarsened echo texture and enlargement of the left lobe and caudate lobe, nodular liver contour and splenomegaly) and endoscopic findings (varices or portal gastropathy). Patients with high blood pressure (BP) at baseline and those with previous history of hypertension were classified as hypertensive patients.

Patients were excluded if they had, at baseline, the presence of other causes of liver disease, positive screening for viral hepatitis B, and HIV, pregnancy or lactation, concomitant disease with reduced life expectancy, heart failure, chronic or acute renal disease, severe psychiatric conditions, drug dependence, presence of secondary causes of hypertension that were screened according to recommended guidelines [21] and evidence of liver cancer on the basis of ultrasonography, α-fetoprotein (AFP) levels higher than 200 ng/L, dynamic computed tomography and/or dynamic-contrast magnetic resonance, and/or cytological or histological diagnosis when indicated. A history of alcohol intake (more than 80 g/day for males and more than 60g/day for females) for more than 10 years was recorded, and alcohol abstinence was monitored at each clinic visit in the course of a patient interview and confirmed by relatives. The diagnosis of hypertension was made according to published guidelines. [21].

### Clinical and Laboratory Assessment

All patients were monitored for clinical, biochemical, and hematological status at baseline. Baseline systolic blood pressure (SBP), diastolic blood pressure (DBP), mean arterial pressure (MAP = DBP+[0.333 (SBP-DBP)] and heart rate (HR) were assessed at baseline. At this time, three measurements of clinic blood pressure were taken at 10-min intervals by a healthcare professional trained in the measurement of blood pressure. Blood pressures were measured after at least 10 minutes in the seated position using an appropriately sized cuff and mercury sphygmomanometer. The three clinic BP measurements were averaged. Any history of arterial hypertension was recorded at baseline, and the administration of the anti-hypertensive drugs was standardized according to recommended guidelines. [22], [23].

After inclusion, patients were followed every 2 months, and each visit included physical examination (including BP measurements), blood tests, and abdominal ultrasound. Pharmacologically treated hypertensive patients who developed normotension (>90/60 and <140/90) during the follow-up were closely monitored with weekly BP readings. The antihypertensive treatment was stopped in those patients who developed hypotension (BP≤90/60 mm/Hg) in three successive readings within a week. Hypotension was diagnosed when the BP fell to ≤90/60 mm/Hg. The development of ascites was the main end point of the study. Development of other decompensation (bleeding and encephalopathy), HCC, and death from any cause were also considered.

Ascites was defined by the presence of signs and symptoms suggestive of ascites on physical examination and was always confirmed on echography or paracentesis.

Upper gastrointestinal bleeding secondary to portal hypertension and hepatic encephalopathy and spontaneous bacterial peritonitis were diagnosed and treated according to established guidelines. [24]–[26] Liver ultrasonography and serum α-fetoprotein determinations were carried out at baseline and every 24 weeks during the study for screening hepatocellular carcinoma. Patients with elevated AFP levels and/or new lesions suspected or detected during ultrasound examination were further evaluated with contrast-enhanced studies such as computed tomography or magnetic resonance and/or echo-guided needle liver biopsy. The treatment of hepatocellular carcinoma was implemented according to recommended guidelines. [27].

The antiviral treatment was implemented following the recommended guidelines, commensurate with available resources in our country.

During the period study, patients with medium-large varices received prophylactic nonselective β-blockers (NSBB) based on the recommended guidelines. [28] Patients started with a dose of propranolol 40 gm/day given orally twice a day and it was increased up to achieve a maximally tolerated dose or until a heart rate of approximately 55 beats/min. Patients with hypotension (≤90/60 mm/Hg) were not treated with NSBB. The treatment with NSBB was discontinued in those subjects who developed hypotension during the follow-up.

The HCV-RNA level was quantified by PCR assay (Amplicor Monitor HCV v.2.0; Roche Molecular System; lower limit of detection, 600 IU/ml). HCV genotyping was performed by reverse hybridization (Inno-LiPA HCV; Innogenetics, Ghent, Belgium).

### Definition of Outcomes

Our study describes the occurrence of ascites at 312 weeks of follow-up. The occurrence of variceal hemorrhage or hepatocellular carcinoma (HCC) may increase the risk of development of ascites; therefore, they were considered as competing risk events at the same time when they occurred. Moreover, deaths, either related or unrelated to liver disease, and liver transplantation were also analyzed as competing risk events.

Patients were followed until the end of the study, development of ascites or any event indicating disease progression (variceal bleeding, HCC or liver transplantation), or death. Patients lost to follow-up were censored at the last date known to be alive.

All clinical outcomes were verified and confirmed by three expert hepatologists when they occurred.

The study was conducted in compliance with the Declaration of Helsinki and approved by the ethics committee and the institutional review board of the National Institute of Gastroenterology. All patients provided written informed consent for participation.

### Statistical Analysis

The baseline characteristics were summarized in percentage for categorical variables and as medians and interquartile range (IQR) for continuous variables. The chi-square test was applied to categorical variables and the Wilcoxon signed-ranks test to compare medians between groups.

The main objective of the study was to explore the potential association between arterial BP and the development of ascites. To estimate effects of covariates on ascites development, univariable and multivariable competing risk regression models for the subdistribution hazards were performed according to the method of Fine and Gray. [29], [30] The strength of the association between each predictive variable and the outcome was assessed using the subhazard ratio (sHR) with 95% confidence intervals (CI) (see Text S1).

Variables that were significant (p<0.15) in univariable analysis and those known as weighted prognostic indicators were included in multivariable analysis.

Covariates were selected for the analyses according to their biologically plausible potential to act as confounders or predictors for the outcome.

The probable prognostic predictors, including age, gender, previous history of alcohol intake, presence of varices, antihypertensive drugs, NSBB for portal hypertension, MAP, bilirubin, AST/ALT ratio, creatinine, albumin, platelets, INR for prothrombin time (PT), and serum sodium were considered for overall analyses. The cardioselective and nonselective beta-blockers were entered separately into the model, and the influence of NSBB on ascites development was assessed, including data of patients receiving beta-blockers because of portal hypertension. In order to explore the effect of different cutoffs of MAP on the probabilities of ascites development, four groups of approximately equal size were created (quartiles labeled MAP>100.31 mm/Hg, MAP between 93.32 and 100.31 mm/Hg, MAP between 83.32 and 93.32, and MAP<83.32 mm/Hg). [31] Thus, unadjusted and adjusted cumulative probabilities of ascites development according to these cutoffs were calculated using cumulative incidence methods. [29].

The assessments of proportional assumptions were visually inspected using Schoenfeld-type residuals. The assumptions of proportionality were met both globally (overall model) and individually for each predictor variable.

The missing values were imputed by applying multiple imputations method in which missing data are imputed or replaced with a set of plausible values. [32], [33].

In this study there were 4 events (ascites) for each covariate evaluated. Overall, patients were included in an intention-to-treat analysis.

All confidence intervals, significance tests, and resulting *P* values were two-sided, with an alpha level of 0.05.

Statistical analyses were performed using STATA software, release 11.

## Results

**Figure 1:**
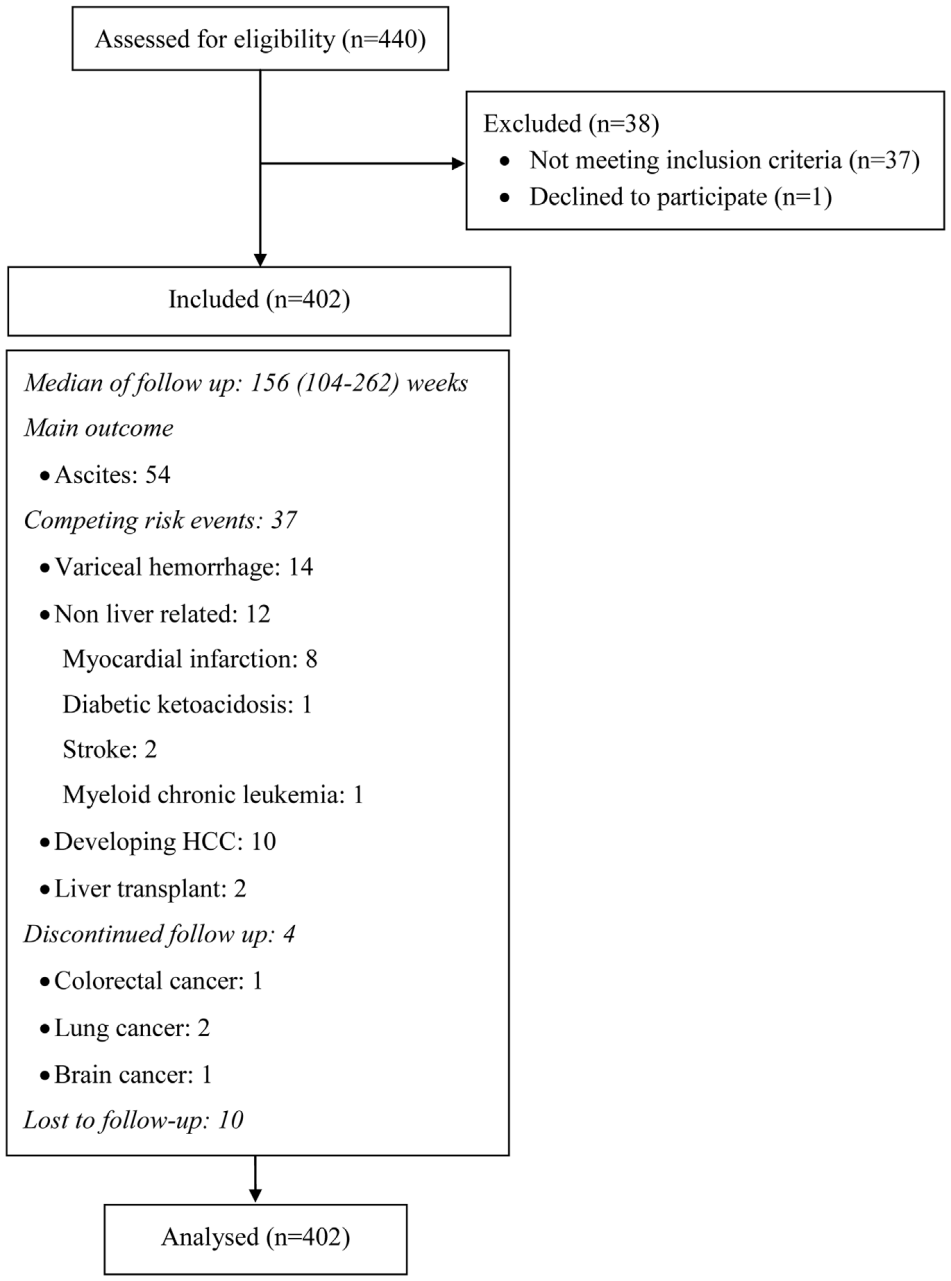
Flow of patients through the study.

A total of 440 patients were examined for eligibility, and 402 were included in the study. Thirty eight patients were excluded during the screening period because they met one or more of the exclusion criteria at baseline: 7 patients with inexact date of diagnosis of cirrhosis at baseline, 30 subjects with history of hepatic decompensation and 1 individual withdrew their consent. No patient was excluded due to secondary hypertension. The median follow-up period was 156 weeks (IQR, 104–262 weeks) overall. The flow of the participants through the study is presented in Figure 1.

At baseline, 163 patients (41%) had a current history of essential arterial hypertension based on high BP levels (systolic BP≥140 mm/Hg and/or diastolic BP≥90 mm/Hg on at least 3 occasions) without antihypertensive medication or normal BP levels with medication. Of these, 148 were receiving antihypertensive therapies at the protocol admission. Diuretics and ACE inhibitors were the most commonly used antihypertensive drugs. Among hypertensive patients, 28 (17%) developed hypotension over time and the treatment was discontinued. One hundred and eight individuals had varices at baseline. Of them, 61 had medium or large varices and 54 were treated with NSBB. Seven patients were treated with endoscopic variceal ligation due to hypotension (≤90/60 mm/Hg). During the follow-up, overall alcohol users became abstainers.

Among patients who developed ascites during the follow-up, 42 of 54 (78%) were women and 12 of 54 (22%) were men. The proportion of patients included in the Child-Pugh B class and the MELD score medians were significantly higher in patients who developed ascites. Patients with ascites development showed significantly lower baseline systolic, diastolic and mean arterial BP than those without ascites. The mean arterial pressure (MAP) was significantly lower in patients with ascites development (75.9 mm/Hg [95%CI, 70.3–84.3]) than those without ascites (93.6 mm/Hg [95% CI: 86.6–102.3]). In contrast, there were no differences in heart rates between both groups.

Among patients with ascites, higher values of AST/ALT ratio, total bilirubin and INR were recorded as compared to subjects without ascites. Likewise, lower levels of platelets and albumin were found in patients who developed ascites.

Most patients were naïve to antiviral treatment during the study period. During the follow-up period, 182 patients underwent antiviral therapy, 160 completed the antiviral treatment, and 31 achieved an SVR (10 of 48 subjects who completed treatment with Peg/Rib and 21 of 112 patients who completed treatment with IFN/Rib). The SVR rates were significantly lower in those receiving Peg/Rib. Most of the patients treated with Peg/Rib were previous non responders to IFN/Rib when they were treated at pre-cirrhotic stages, thus, the SVR may be lower. Finally among subjects with SVR, only one developed an episode of ascites eight months after concluding the antiviral therapy. No patient developed ascites or other episodes of hepatic decompensation during the antiviral treatment.

Table 1 summarizes the baseline characteristics of patients enrolled in the study.

**Table 1:** Baseline characteristics according to ascites development during the follow-up.

Variables	Overall N = 402	Development of ascites	
		Yes n = 54	No n = 348
Age (y)	59 (50–65)	58 (50–63)	59 (50–66)
Sex, n (%)			
Female	244 (61%)	42 (78%)	202 (58%)
Male	158 (39%)	12 (22%)	146 (42%)
Diagnosis of cirrhosis, n (%)			
Liver biopsy	305 (76%)	41 (76%)	264 (76%)
Other criteria	97 (24%)	13 (24%)	84 (24%)
HCV RNA >600,000 IU/ml	348 (87%)	48 (89%)	300 (86%)
HCV genotype 1, n (%)	385 (96%)	53 (98%)	332 (95%)
Gastroesophageal varices, n (%)	108 (27%)	12 (22%)	96 (28%)
Child-Pugh score, n (%)			
A	338 (84%)	29 (54%)	309 (89%)
B	64 (16%)	25 (46%)	39 (11%)
C	0 (0%)	0 (0%)	0 (0%)
MELD score	9 (8–12)	11 (10–14)	9 (7–10)
Arterial hypertension, no (%) *	163 (41%)	8 (15%)	155 (45%)
Antihypertensive drugs, n (%)	148 (37%)	10 (19%)	138 (40%)
Diuretics	86 (21%)	4 (7%)	82 (24%)
ACE inhibitors	61 (15%)	2 (4%)	59 (17%)
Cardioselective β-blockers	22 (6%)	4 (7%)	18 (5%)
Calcium antagonists	18 (5%)	1 (2%)	17 (5%)
Systolic blood pressure (mm/Hg)	122 (112–139)	105 (95–112)	123 (115–140)
Diastolic blood pressure (mm/Hg)	79 (70–82)	61 (59–71)	80 (71–83)
Mean arterial pressure (mm/Hg)	93.3 (83.3–100.3)	75.9 (70.3–84.3)	93.6 (86.6–102.3)
Heart rate (b/min)	76 (66–80)	75 (65–84)	76 (67–80)
Alcohol intake, n (%)	54 (13%)	6 (11%)	48 (14%)
Nonselective β-blockers, n (%) †	54 (13%)	6 (11%)	48 (14%)
Median doses	30 (20–50)	40 (40–80)	30 (30–60)
AST/ALT ratio	1.11 (0.88–1.35)	1.20 (0.95–1.42)	1.09 (0.87–1.34)
Creatinine (mmol/L)	78 (68–88)	71 (63–85)	79 (68–88)
Total bilirubin (mmol/L)	17.4 (13–24)	20 (16.1–35)	16.7 (12.2–21.7)
Albumin (g/L)	40 (37–42.7)	38.6 (35–41)	40 (37–43)
INR for prothrombin time	1.20 (1.1–1.4)	1.41 (1.29–1.59)	1.19 (1.08–1.31)
Sodium, mEq/L	143 (140–145)	142.1 (140–144.5)	143 (140.5–145)
Platelets (x 10^9^/L)	142 (111–180)	105 (81–144)	144 (115–180)
α-fetoprotein (ng/ml)	11 (6–17)	12 (8–16)	11 (7–15)

Abbreviations: MELD, Model for End-Stage Liver Disease; ALT, alanine aminotransferase; AST, aspartate aminotransferase; INR, international normalized ratio.

* Arterial hypertension included patients with current history of hypertension and antihypertensive medication or high blood pressure (systolic ≥140 mm/Hg and/or diastolic ≥90 mm/Hg on at least 3 occasions) without antihypertensive drugs.

† Nonselective beta-blockers as primary prophylaxis for variceal bleeding.

For all laboratory measures and for continuous demographics: Wilcoxon signed-ranks tests.

Proportions: percentage, P value chi-square.

Quantitative data were expressed as median (25%–75% quantiles).

The Child-Pugh and MELD scores are measures of the severity of liver disease.

### Development of Ascites

During follow-up, 54 patients (13%) developed ascites. The 52-, 104-, 156-, 208-, 260- and 312-week cumulative incidences were 2%, 3%, 5%, 7%, 11% and 14%, respectively.

**Figure 2:**
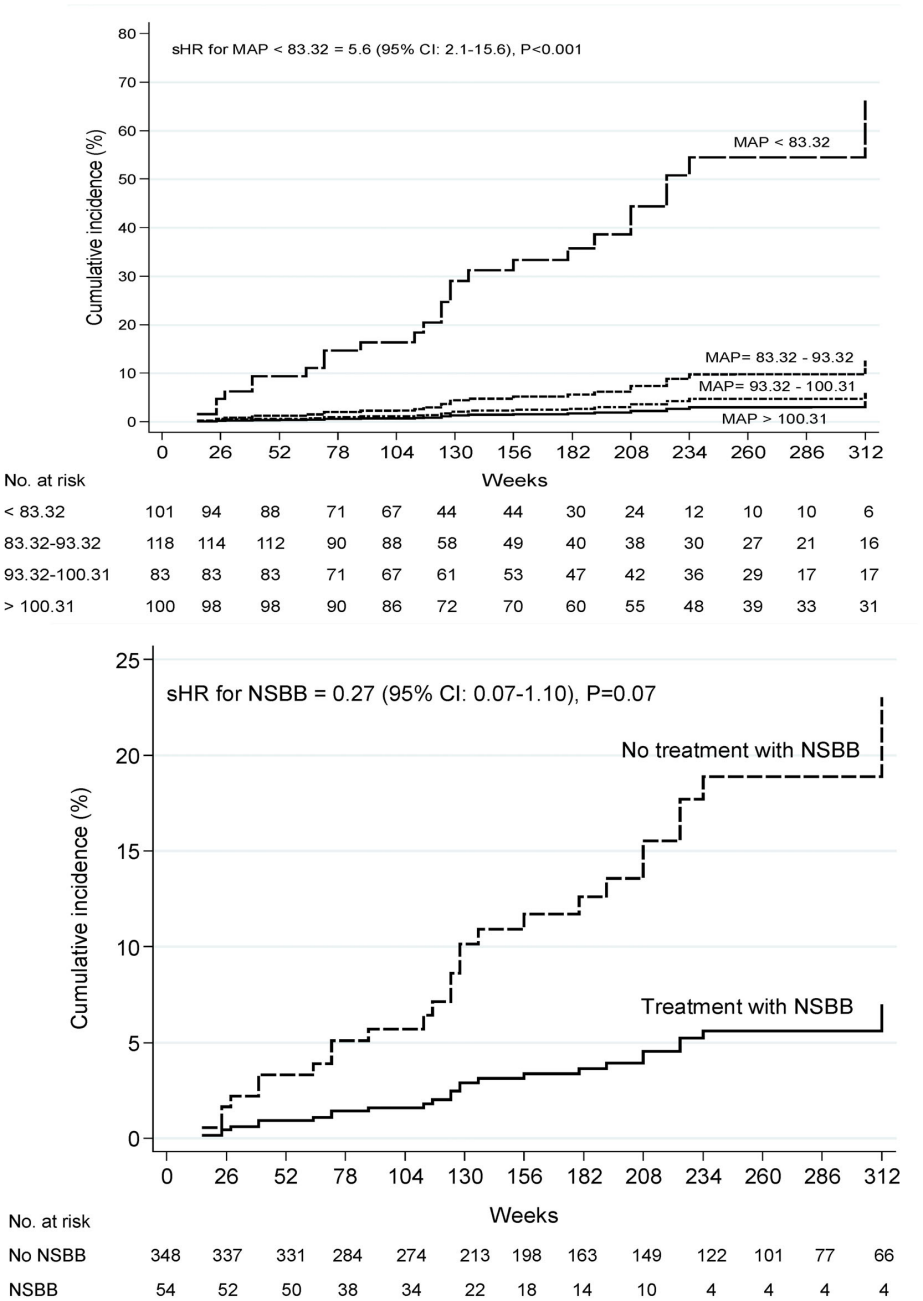
Unadjusted cumulative probabilities for ascites development. (A) Cumulative probabilities of ascites according to different cutoffs of mean arterial pressure*. (B) Cumulative probabilities of ascites based on the use of nonselective β-blockers. * Four groups of approximately equal size were created by MAP (quartiles labeled MAP>100.31 mm/Hg, MAP between 93.32 and 100.31 mm/Hg, MAP between 83.32 and 93.32, and MAP<83.32 mm/Hg). Abbreviations: MAP, mean arterial pressure; NSBB, nonselective β-blockers.

**Figure 3:**
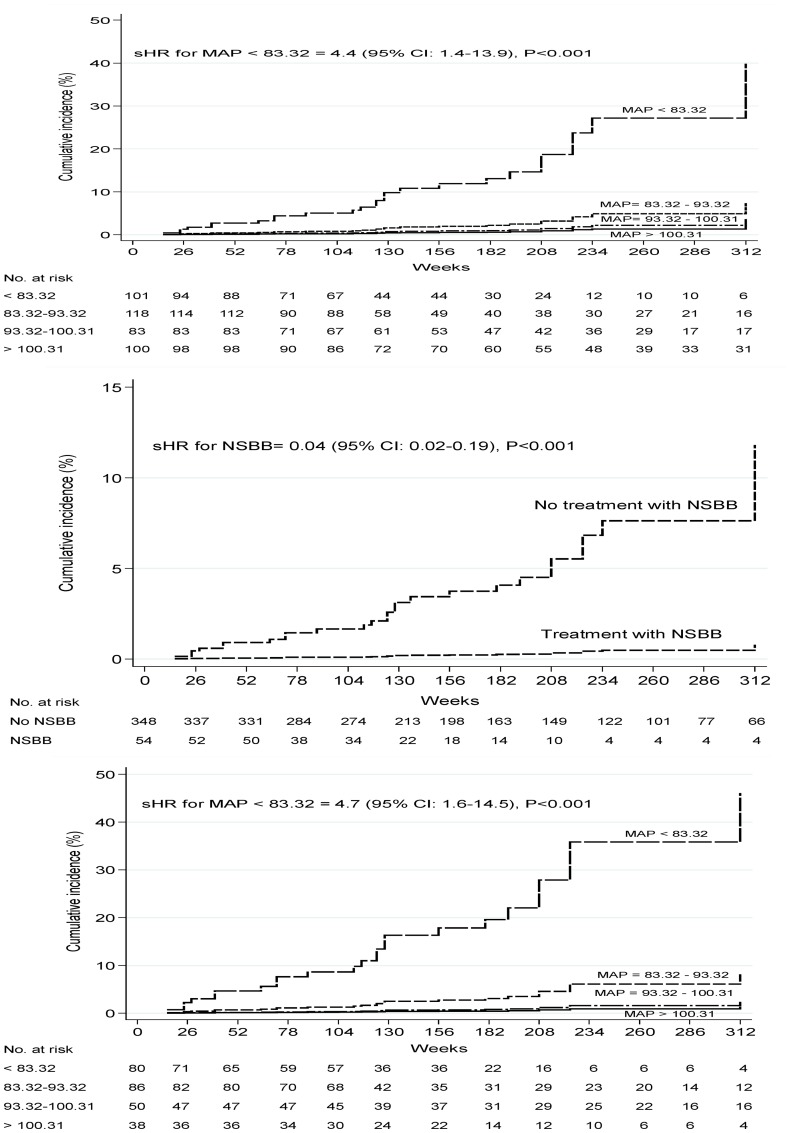
Adjusted* cumulative probabilities for ascites development. (A) Cumulative probabilities of ascites according to different cutoffs of MAP^†^. (B) Cumulative probabilities of ascites based on the use of nonselective β-blockers. (C) Cumulative probabilities of ascites according to different cutoffs of MAP* in a subgroup of patients without antihypertensive drug therapy. * Adjusting covariates for MAP were gender, presence of varices, antihypertensive drugs (diuretics, ACE inhibitor and calcium antagonist), NSBB for portal hypertension, bilirubin, AST/ALT ratio, creatinine, albumin, platelets, INR for prothrombin time, and serum sodium, and for NSBB were the same including MAP. ^†^ Four groups of approximately equal size were created by MAP (quartiles labeled MAP>100.31 mm/Hg, MAP between 93.32 and 100.31 mm/Hg, MAP between 83.32 and 93.32, and MAP<83.32 mm/Hg). Abbreviations: MAP, mean arterial pressure; NSBB, nonselective β-blockers.

As shown in the Figures 2A and 3A, CIs for ascites development at 312 weeks (equivalent to 6 years) were considerably higher in patients with MAP<83.32 mm/Hg than those with higher values of MAP. After adjusting for covariates, the cumulative probability of ascites was 40% (95%CI, 34%–48%) for patients with MAP<83.32 mm/Hg. In contrast, 6-years CIs of ascites were almost similar among patients with MAP values between 83.32 mm/Hg and 93.32 mm/Hg (7% [95% CI: 4%–12%]), between 93.32 mm/Hg and 100.31 mm/Hg (5% [95% CI: 4%–11%]) or higher than 100.31 mm/Hg (3% [95% CI: 1%–6%]). In order to confirm the potential effects of different cutoffs of MAP on the risk of ascites development, a further analysis was performed in the cohort of patients free of an antihypertensive regimen. To do that, 148 pharmacologically hypertensive treated patients were excluded from this analysis. Therefore, 254 subjects were stratified according to predefined cutoffs of MAP and the cumulative occurrence of ascites was assessed overtime. After adjusting for covariates, the probability of developing ascites was almost similar to the overall group of patients (46%; 95% CI: 37%–52%). The cumulative incidence of ascites was considerably lower in patients with MAP cutoffs >83.32. The probability of developing ascites in patients with MAP cutoffs higher than 83.32 was almost similar to the overall cohort of patients. Similarly, 6-year CIs of ascites were similar among patients with MAP values between 83.32 mm/Hg and 93.32 mm/Hg (8% [95% CI: 5%–13%]), between 93.32 mm/Hg and 100.31 mm/Hg (3% [95% CI: 2%–9%]) or higher than 100.31 mm/Hg (2% [95% CI: 1%–7%]) (Figure 3C).

Figures 2B and 3B show unadjusted and adjusted CIs for ascites development in patients treated or not with NSBB for portal hypertension. Patients treated with NSBB showed cumulative lower rates of ascites (2%, 95% CI: 1%–8%) as compared to patients without this medication (12%, 95% CI: 8%–22%) (Figure 3B).

### Predictors of Ascites Development

By multivariate analysis, mean arterial pressure (MAP), gender, presence of gastroesophageal varices at baseline, use of NSBB as primary prophylaxis for variceal bleeding, AST/ALT ratio, platelets count and INR were independently associated with the occurrence of ascites (Table 2). The estimated sHR for ascites development increased in 4.4 (95% CI, 1.4–13.9) times for subjects with lower values of MAP (<83.32) (Figure 3A). After adjusting for covariates, the sHR of developing ascites in the subgroup of patients without antihypertensive drug therapies and MAP<83.32 was increased in 4.7 fold (Figure 3C). Conversely, the risk of ascites development was almost comparable for higher MAP cutoffs. Subhazard ratios of 0.14, 0.08 and 0.04 for higher MAP cutoffs mean that there were 86%, 92% and 96% less risk for ascites development; however, there were no statistical differences among these groups (Figure 3A). After excluding patients with antihypertensive treatment, the risk of developing ascites in patients with MAP>83.32 was consistently lower and almost similar in all predefined quartiles (Figure 3C). Subhazard ratios of 0.14, 0.03 and 0.02 were estimated for MAP cutoffs of 83.32–93.32, 93.32–100.31 and >100.31, respectively. There was no statistical difference between these groups.

**Table 2:** Variables found as significant predictors of ascites development.

Variable	Development of ascites n = 54			
	Univariable		Multivariable	
	sHR	*P*	sHR (95% CI)	*P*
Age (years)	0.98	0.30	–	–
Gender (male)	0.49	0.03	0.33 (0.18 to 0.57)	<0.001
Gastroesophageal varices (yes)	2.62	0.001	4.36 (2.12 to 8.61)	<0.001
Alcohol intake (yes)	1.15	0.56	–	–
Antihypertensive drugs				
Diuretics (yes)	0.34	0.01	–	–
ACE inhibitors (yes)	0.25	<0.01	–	–
Cardioselective β-blockers (yes)	0.71	0.48	–	–
Calcium antagonists (yes)	0.58	0.05	–	–
MAP*	0.88	<0.001	0.90 (0.86 to 0.94)	<0.001
MAP<83.32 mm/Hg	–	–	–	–
MAP 83.32–93.32 mm/Hg	0.19	<0.001	0.14 (0.06–0.38)	<0.001
MAP 93.32–100.31 mm/Hg	0.10	<0.001	0.08 (0.03–0.29)	<0.001
MAP>100.31 mm/Hg	0.06	<0.01	0.04 (0.02–0.19)	<0.001
Nonselective β-blockers † (yes)	0.27	0.07	0.04 (0.01 to 0.15)	<0.001
AST/ALT ratio	1.66	0.08	3.05 (1.22 to 7.64)	0.01
Albumin	0.91	<0.001	–	–
Creatinine	0.98	<0.01	–	–
INR for prothrombin time	7.73	<0.001	11.4 (4.7 to 27.4)	<0.001
Platelets	0.98	<0.001	0.98 (0.97 to 0.99)	0.04
Serum sodium	0.92	0.01	–	–
Total bilirubin	1.04	<0.001	–	–

Results based on competing risk regression models.

Abbreviations: CI, confidence interval; sHR, subHazard ratios; INR, international normalized ratio.

*Overall MAP and their quartiles were analyzed separately in different multivariable models.

† Non selective beta-blockers as primary prophylaxis for variceal bleeding.

## Discussion

The present study clearly demonstrates that HCV-related compensated cirrhotic patients with low blood pressures, defined as a MAP<83.32 mm/Hg, have an increased probability of ascites development at 6 years of follow-up. These data suggest that the presence of hypotension in cirrhotic patients may reflect an accentuation in the degree of arterial vasodilation and circulatory dysfunction, with a subsequently increased risk of ascites development.

In our study, the prevalence of hypertension was higher than rates reported in the general population. However, existing data suggest that the prevalence of hypertension consistently increases with age. A particularly high prevalence is reported in people aged 40 years and older (range of 42% to 73%). [34] In the present study, 400 (99%) patients were older than 40 years. On the basis of these estimates, our prevalence rate corresponds to that reported in people older than 40 years.

In the most studies, the prevalence of essential hypertension in cirrhotic patients has been reportedly very low (range, 3–7%). [20], [35], [36] However, these prevalence rates have been calculated based on blood pressure levels of patients without antihypertensive therapies, and a higher proportion of decompensated patients, in whom hypotension is common, were included.

Although the prevalence of essential hypertension was notably higher in our study, only 9% of patients had a diagnosis of hypertension without medication. Furthermore, most of the patients enrolled in our study were classified as Child-Pugh A.

A recent report has hypothesized that arterial hypertension may protect cirrhotic patients from complications attributable to circulatory dysfunction. [20] This study reported that cirrhotic patients with arterial hypertension have no signs of overall peripheral vasodilation as compared to normotensive counterparts; therefore, they are less prone to develop vasodilatory complications such as hepatorenal (HRS) and hepatopulmonary (HPS) syndromes. With the purpose of exploring the effect of different values of MAP on the risk of developing ascites, we created 4 prognostic groups of approximately equal size (quartiles) in the overall cohort of patients. A subsequent analysis was performed to determine the risk of different MAP cutoffs in a subgroup of patients who were not taking antihypertensive therapy. Our results indicate a notable reduction in ascites development in the three groups of patients with MAP higher than 83.32 mm/Hg. However, there were no statistically significant differences between these groups. The probability of developing ascites was similar in patients with MAP cutoffs ranging from normal to high blood pressure. Thus, our results do not support the hypothesis that high blood pressures should protect from the development of ascites. However, further prospective and long-term studies are needed to evaluate whether high blood pressure reduces the incidence of HRS or HPS in decompensated patients with either ascites or more profound circulatory dysfunction.

The mean arterial pressure was one of the most consistent and independent predictors of ascites development. Thus, this variable should be taken into account in the prediction of ascites development in cirrhotic patients.

Many known factors such as the presence of gastroesophageal varices, INR, gender, platelets count and AST/ALT ratio were independent predictors of developing ascites. Although the benefit of SVR on major clinical outcomes in patients with CHC has been recognized in several studies, [37]–[40] its impact on ascites development was not analyzed in the current study. Our study was not intended to evaluate the effect of SVR on the occurrence of ascites. In fact, most patients were naïve to antiviral treatment during the study period and only 182 patients underwent antiviral therapy. The beneficial effects of SVR on major clinical outcomes should be evaluated in the context of randomized clinical trials.

Another important finding of this prospective study was the finding that the long-term use of NSBB as primary prophylaxis for variceal bleeding was a protective factor against ascites development. This probably indicates that the majority of patients were excellent responders to β-blocker treatment; however, the hepatic venous pressure gradient was not measured to confirm this assertion. In support of our results, a recent study has suggested that treatment with nadolol (NSBB) in cirrhotic patients with high-risk varices can delay the occurrence of a first event of ascites. [41] Those patients who reached a hemodynamic response were not only at reduced risk of first variceal bleeding, but also for developing ascites. Other studies have also previously confirmed the effect of NSBB on the reduction of developing ascites. [42], [43].

This encouraging result should be carefully interpreted because our study was not a randomized controlled trial (RCT), which is the best way to evaluate the effects of specific medications.

Further studies may confirm the relationship among arterial blood pressures and the development of ascites in cirrhotic patients with different etiologies. Moreover, a longer follow up is required to confirm or dismiss the protective effect of high blood pressures on the occurrence of ascites or other complications related to circulatory dysfunction.

In conclusion, the presence of low BP among compensated cirrhotic patients is an indirect marker of the degree of circulatory dysfunction, which is linked to an elevated risk of ascites development; therefore, more stringent follow-up may be implemented in these patients. The presence of high BP in compensated cirrhotic patients is not a long-term protective factor for ascites development. In general, other predictors for ascites development should reflect the degree of portal hypertension and hepatocellular dysfunction. Further randomized controlled trials are needed to determine the effect of NSBB on ascites development, in particular in patients with high-risk varices.

## Supporting Information

Text S1 Supplementary material for competing risk analysis*. *The competing risk analysis performed in the study is detailed in this section.
(DOC)
