# Supplementary material for: Arterial Blood Pressure Is Closely Related to Ascites Development in Compensated HCV-Related Cirrhosis
Source: PLoS One. 2014 Apr 22;9(4):e95736. doi: 10.1371/journal.pone.0095736 (PMC3995756; doi:10.1371/journal.pone.0095736)
Supplement: Text S1 — Supplementary material for competing risk analysis*. *The competing risk analysis performed in the study is detailed in this section. (DOC) [file pone.0095736.s001.doc]

Supplementary material for competing risk analysis

A competing risk is defined as an event whose occurrence precludes or alters the probability of occurrence of a main event under examination. In this setting, the appropriate estimate of the probability of failure is described by the so-called cumulative incidence.

In the present study, the presence of variceal hemorrhage or HCC was considered as competing risk events at the same time when they occurred. Moreover, deaths, either related or unrelated to liver disease, and liver transplantation were also analyzed as competing risk events.

In our study, the cumulative incidences method were applied to compute the event probabilities for the main outcome (ascites development) in the presence of competing risk events, because of Kaplan-Meier estimator treats inadequately competing events as if they were censored, and consequently overestimates the cumulative event-specific probability.

Cumulative incidence is defined as the cumulative probability of event k having occurred in the presence of other competing events, which prevent occurrence of the event of interest. In order to assess the associations between baselines covariates and the main outcome (ascites development), univariable and multivariable competing risk regression models for the subdistribution hazards were performed according to the method of Fine and Gray. This method allows direct assessment of baseline covariates on the cumulative incidence function and makes use of the hazard of subdistribution which is a function of the cumulative incidence for the corresponding cause of failure (i.e., subjects with a lower subdistribution hazard also have a lower cumulative incidence function). The strength of the association between each predictor variable and the outcome was assessed using the subhazard ratio, which is the ratio of hazards associated with the cumulative incidence function (CIF) in the presence of and in the absence of a prognostic factor. Subhazard ratios can be interpreted similarly to hazard ratios in Cox regression models. We prefer to use this model over a cause-specific Cox hazard model, which is difficult to interpret when the overall effects of covariates on the cumulative incidence functions are the interest.

**References**

1. Pintilie M (2007) Analysing and interpreting competing risk data. Stat Med 26: 1360-1367.

2. Grunkemeier GL, Jin R, Eijkemans MJ, Takkenberg JJ (2007) Actual and actuarial probabilities of competing risks: apples and lemons. Ann Thorac Surg 83: 1586-1592.

3. Satagopan JM, Ben-Porat L, Berwick M, Robson M, Kutler D, et al. (2004) A note on competing risks in survival data analysis. Br J Cancer 91: 1229-1235.

4. Putter H, Fiocco M, Geskus RB (2007) Tutorial in biostatistics: competing risks and multi-state models. Stat Med 26: 2389-2430.

5. Dignam JJ, Kocherginsky MN (2008) Choice and interpretation of statistical tests used when competing risks are present. J Clin Oncol 26: 4027-4034.

6. Dignam JJ, Zhang Q, Kocherginsky M (2012) The use and interpretation of competing risks regression models. Clin Cancer Res 18: 2301-2308.
